# A Novel *ANO1* Gene Variant is Associated with Intestinal Dysmotility Syndrome Masquerading as Hirschsprung Disease: A Case Report

**DOI:** 10.1097/PG9.0000000000000317

**Published:** 2023-05-09

**Authors:** Ahmed H. Al Sharie, Balqis M. Abu Mousa, Yazan O. Al Zu’bi, Mohammad A. Al Qudah, Saied A. Jaradat, Ahmad Barakat, Eyad Altamimi

**Affiliations:** From the * Faculty of Medicine, Jordan University of Science & Technology, Irbid, Jordan; † Faculty of Medicine, Hashemite University, Zarqa, Jordan; ‡ Department of Biotechnology and Genetic Engineering, Jordan University of Science and Technology, Irbid, Jordan; § Department General Surgery and Urology, Faculty of Medicine, Jordan University of Science and Technology, Irbid, Jordan; ∥ Department of Pediatrics and Neonatology, Faculty of Medicine, Jordan University of Science and Technology, Irbid, Jordan.

**Keywords:** intestinal dysmotility syndrome, ANO1, novel mutation

## Abstract

Anoctamin 1 (ANO1)-related intestinal dysmotility syndrome (OMIM: 620045) is an extremely rare disorder with only 2 cases reported in the medical literature. We present the clinical scenario of a 2-month-old male infant that presented to our center with diarrhea, vomiting, and abdominal distension. Routine investigations did not yield a clear diagnosis. Whole-exome sequencing showed a novel homozygous nonsense *ANO1* pathogenic variant (c.1273G>T) with a protein alternation of p.Glu425Ter that fits the patient’s phenotype. Sanger sequencing revealed the same *ANO1* variant in both parents in a heterozygous form confirming an autosomal recessive mode of inheritance. The patient experienced multiple bouts of diarrhea-related metabolic acidosis, dehydration, and severe electrolyte imbalances that required intensive care unit monitoring. The patient was managed conservatively and being followed regularly in an outpatient setting.

## INTRODUCTION

Peristalsis is a fundamental physiological phenomenon found to maintain food propulsion through the gastrointestinal tract. Over the past decade, many supreme discoveries were established regarding the molecular mechanisms controlling peristalsis as in mechanotransduction and chemotransduction. Worth mentioning, the gastrointestinal tract is the only hollow organ with a confined nervous system known as the enteric nervous system that can function independently even with the lack of central nervous system input (1). The gut’s pacemaker that orchestrates classical rhythmic motion are the interstitial cells of Cajal. These cells induce phasic contractions through continuous slow waves generation, alluding to a connection between the enteric nervous system and smooth muscles (2). Their mode of action is a multilayered complex notion that is being extensively studied; discerning activators, inhibitors, modifiers, and modulators. Recently, the elusive role of anoctamin 1 (ANO1 or TMEM16A) in gastrointestinal motility has become more rectified (3). ANO1 is a calcium-activated chloride channel that is highly expressed in the interstitial cells of Cajal (4) with preferential use over c-kit in Hirschsprung disease histopathological examination (5). It was demonstrated that ANO1 possesses a pivotal function in generating slow waves with no apparent effect on interstitial cells of Cajal structural integrity as elucidated by knockout mice experiments (6). Such an argument has been supported by overwhelming evidence as previously described (7–9). In this report, we present the clinical scenario of intestinal dysmotility syndrome; a recently discovered disease representing the clinical consequences of ANO1 dysfunction.

## CASE PRESENTATION

**FIGURE 1.**
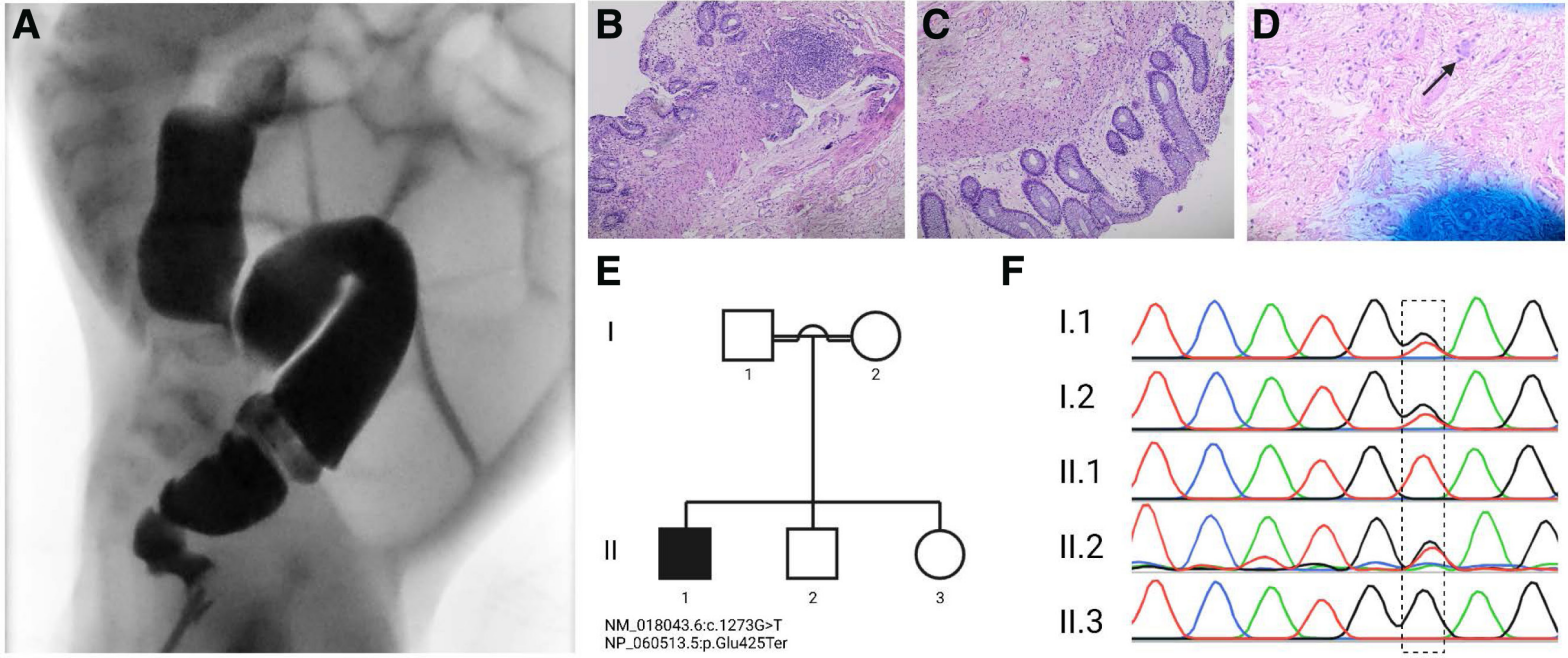
Gastrografin barium enema illustrated a reversed rectosigmoid ratio with the lower rectum being smaller in caliber compared to the sigmoid and the rest of the colon (A). Histopathological examination of the rectal biopsy indicated the presence of colonic ganglionic cells as highlighted by the black arrow (B–D). Pedigree of the affected family (E) and Sanger electropherograms of the family members (F).

A 2-month-old male infant; a preterm (34 weeks of gestation) product of a cesarian section with a history of neonatal intensive care unit admission due to respiratory distress syndrome and delayed passage of meconium until day four of life, presented with diarrhea, vomiting, and abdominal distension for 3 weeks duration. The vomit was nonbloody, nonmucoidal, and yellow in color with a frequency of 1–2 times per day. While the diarrhea was loose, watery, and yellow in color happening 4–5 times a day. Clinical suspicion of galactosemia, cystic fibrosis, and Hirschsprung disease was raised and investigated accordingly. The galactose-1-phosphate uridyltransferase assay and the cystic fibrosis gene sequencing came back negative. Grografin barium enema illustrated a reversed rectosigmoid ratio with the lower rectum being smaller in caliber compared to the sigmoid and the rest of the colon (Fig. 1A). Multiple gas-filled loops were seen in the intestines suggesting a distal intestinal obstruction. All these findings correlate with Hirschsprung disease. Surprisingly, histopathological examination of the rectal biopsy indicated the presence of colonic ganglionic cells (Fig. 1B–D). Since then, the patient experienced multiple bouts of illness with the same features as the initial admission. The suspicion of a genetic disorder was confirmed using whole-exome sequencing (WES). WES presented a novel homozygous nonsense *ANO1* pathogenic variant (c.1273G>T) with a protein alternation of p.Glu425Ter that fits the patient’s phenotype. The parents are consanguineous and Sanger sequencing revealed the same *ANO1* variant in a heterozygous form confirming an autosomal recessive mode of inheritance (Fig. 1E, F). He was admitted multiple times to the pediatric intensive care unit due to diarrhea-related metabolic acidosis and dehydration with multiple severe electrolyte imbalances. He was managed conservatively and followed regularly in an outpatient clinic.

## DISCUSSION

In 2021, Park et al. (10) identified for the first time a pathogenic *ANO1* variant related to fatal neonatal disease in 2 brothers. The first patient was born at term with a complicated pregnancy due to polyhydramnios, he was dysmorphic (broad philtrum, arched palate, and low set ears) with an initial diagnosis of atypical necrotizing enterocolitis. The patient exhibits multiple bouts of illness characterized as projectile vomiting, bloody diarrhea, and distended abdomen with multiple distended intestinal loops on imaging. He was failing to thrive and eventually died from cardiac arrest. His brother was a premature product of a pregnancy also complicated with polyhydramnios. At 3 weeks of age, he developed diarrhea, hypotonia, weight loss, and feeding problems. Later on, bilateral cataracts and developmental delays were noted. Further investigations revealed elevated chloride levels in the sweat chloride test without the typical features of cystic fibrosis. WES revealed a deleterious homozygous variant in the *ANO1* gene (c.897 + 3_897 + 6delAAGT). The pathogenicity of the variant was confirmed through multiple *in vitro* functional experiments aided with *in silico* modeling of the TMEM16A variant. To date, this is the only report describing a pathogenic *ANO1* variant with its clinical complications. To the best of our knowledge, we present the third case of ANO1-related intestinal dysmotility syndrome (OMIM: 620045). Our case seems to represent a milder form of the syndrome in comparison with previous patients in which he was not dysmorphic, was gaining weight normally, and did not have cataracts, hypotonia, or developmental delay. After many bouts of illness, the patient is now stable and being followed in an outpatient clinic regularly. The authors endorse the need of many functional studies to characterize the presented variant for appropriate genotype-phenotype correlation.

## ETHICS APPROVAL AND CONSENT TO PARTICIPATE

This report has been conducted and written in accordance with the ongoing regulations for case reports and case series in the King Abdullah university hospital (KAUH). Case reports are exempted from institutional ethical approval by the institutional review board (IRB). Written informed consent was obtained from the patient guardian for the publication of this report and any associated images.

## ACKNOWLEDGMENTS

The authors acknowledge the generous support from 3 billion (Gangnam-gu, Seoul, South Korea); for running and analyzing the results of whole exome sequencing (Rare Disease Genetic Testing).

Informed patient consent was obtained for this report.
